# Glucose consumption and uptake in HepG2 cells is improved by aqueous extracts from leaves, but not rhizomes, of *Posidonia oceanica* (L.) Delile via GLUT-4 upregulation

**DOI:** 10.1007/s00709-025-02076-8

**Published:** 2025-05-24

**Authors:** Giulia Abruscato, Roberta Tarantino, Manuela Mauro, Roberto Chiarelli, Aiti Vizzini, Vincenzo Arizza, Mirella Vazzana, Claudio Luparello

**Affiliations:** 1 Dipartimento di Scienze e Tecnologie Biologiche Chimiche e Farmaceutiche (STEBICEF), Università di Palermo, Viale delle Scienze, 90128 Palermo, Italy; 2 NBFC, National Biodiversity Future Center, 90133 Palermo, Italy

**Keywords:** Marine angiosperm, Liver cells, Natural product, Glucose-lowering

## Abstract

The endemic Mediterranean seagrass *Posidonia oceanica* is a valuable source of natural bioactive compounds that possess significant therapeutic potential. Here, we examined whether aqueous extracts of rhizomes (RE) and green leaves (GLE) of *P. oceanica* could exert a glucose-lowering effect on the HepG2 cell line, chosen as an in vitro model of liver cells. We assessed glucose uptake and storage, expression levels of GLUT-2 and -4 transporters and the exposure of the latter one at cell surface, as well as modulation of the expression, synthesis and/or activation of the *GLUT2*-transcription factor hepatocyte nuclear factor-1 alpha (HNF1α), and insulin receptor substrate-1 (IRS-1), AKT and protein kinase Cζ (PKCζ), which regulate GLUT-4 translocation. Glucose consumption/uptake and glycogen storage were increased with exposure to GLE alone. Furthermore, at the molecular level GLE-induced upregulation of (i) IRS-1, AKT, and PKCζ activation levels, (ii) GLUT-4 translation levels, and (iii) GLUT-4 exposure on the cell surface. Conversely, GLUT-2 protein was downregulated. Therefore, the application of the aqueous extract of green leaves of *P. oceanica* may be suitable for the development of new treatment agents or dietary supplements for diabetes mellitus acting through GLUT-4 mediated glucose import.

**Supplementary Information:**

The online version contains supplementary material available at 10.1007/s00709-025-02076-8.

## Introduction

In the seas and oceans, which make up three-quarters of the Earth’s surface and are home to an immense biodiversity, chemistry is the preferred mode of communication between organisms. This occurs through the synthesis of unique molecules that play an instrumental role in assisting their producers in the defense and reproduction strategies and environmental adaptation mechanisms. Thus, the marine world represents a very rich and underexploited source of chemodiversity, including compounds with extraordinary potentials that have been used only to a small extent for the design of new drugs, cosmetics and nutraceutics (La Barre 2014).

The seagrass *Posidonia oceanica* (L.) (Delile, 1813; Liliopsida, Alismatales: Posidoniaceae) is a submerged marine angiosperm widely distributed in the Mediterranean Sea. The plant has branched roots, and horizontally or vertically growing rhizomes (modified stems) that serve respectively for anchoring in the substrate and for the formation of groups of 4 to 8 simple leaves in the form of green ribbons (Haznedaroğlu and Akarsu 2009). It forms extensive and dense meadows whose state of health, monitored by structural and functional descriptors, is considered as a key ecological bioindicator of the general environmental quality of coastal water areas (Bellissimo et al. 2020). On the other hand, the deposits of leaves, rhizomes and roots of *P. oceanica* brought in by the swells are continuously accumulated on the banks and produce a large amount of waste that gradually decomposes with the loss of organic matter. The use of fresh and dead *P. oceanica*’s debris as a readily available source of biomass has been the subject of several studies (Cocozza et al. 2011; Ferrández-Gómez et al. 2023; Souii et al. 2023).

It is known that, since ancient times, seagrasses have been used as food for coastal populations and as traditional herbal remedies for various pathologies, such as skin problems, sore throats, acne, and joint pain (Batanouny 1999; El-Mokasabi 2014), reason why more recent interest has been focused on elucidating their phytochemical composition and biological activities responsible for their medicinal properties (Kim et al. 2021).

The beneficial properties for human health offered by *P. oceanica*’s extracts have been studied in in vitro cell model systems. For example, the anti-proliferative activity on a panel of different cancer cell lines was reported by Farid et al. (2018). On the other hand, the anti-metastatic potential of the preparations has been suggested by their ability to reduce the expression and activity of gelatinases, enzymes that facilitate cell motility by degrading the extracellular matrix, and to induce autophagy-dependent locomotory inhibition in human HT1080 fibrosarcoma and SH-SY5Y neuroblastoma cells (Barletta et al. 2015; Leri et al. 2018; Vasarri et al. 2021a). Notably, the extract-mediated activation of the autophagic process was also found to reduce lipid accumulation in HepG2 cells (Vasarri et al. 2021b). Regarding its anti-inflammatory role, in LPS-treated RAW 264.7 macrophages the preparation was able to prevent NF-κB activation through the modulation of the ERK1/2 and AKT signaling pathways, downregulating the production of both reactive oxygen species and NO through the reduction of iNOS and COX-2 levels (Vasarri et al. 2020a). Furthermore, exposure of cultured fibroblasts to *P. oceanica*’s extract provided significant protection against oxidative stress and mortality associated with UV exposure, thus confirming its activity against photodamage and free radical production (Messina et al. 2021).

It should be noted that the decoction of *P. oceanica*’s leaves has long been used as an anti-diabetic preparation by people in the coastal areas of Western Anatolia. Consistently, an early report by Gokce and Haznedaroglu (2008) demonstrated its concentration-dependent hypoglycemic and vasoprotective effect when administered orally to alloxan-induced diabetic rats. Vasarri et al. (2020b) demonstrated in vitro the strong anti-glycation capacity of *P.oceanica*’s leaf extract. The butanol extract of *P. oceanica*’s rhizomes encapsulated in gelatin nanoparticles was found to be effective in reversing the hyperglycemic state and improving insulin resistance in diabetic rats, also inducing an increase in GLUT-4 transporter levels (Ammar et al. 2021). Recently, Morresi et al. (2022) reported that the extract from *P. oceanica*’s leaves decreased intracellular accumulation of GLUT2 glucose transporter in differentiated human intestinal CaCo-2 cells.

Diabetes mellitus, a complex metabolic disorder characterized by persistent hyperglycemia and insulin resistance, poses a major threat to human health, as with lifestyles changes and the increase in obesity its prevalence is steadily augmenting worldwide (Goyal et al. 2024). There are many pharmacological resources available to help control blood glucose, but their use causes a number of side effects. On the other hand, as reviewed by Rahman et al. (2022), the development of antidiabetic compounds from plants has attracted much attention due to their lower cost, reduced side effects and greater accessibility; nevertheless, rigorous scientific validation is essential for their medicinal use.

We have previously characterized the different polyphenolic and proteomic compositions of the water-soluble extracts obtained from either green leaves (GLE) and rhizomes (RE) of *P. oceanica*’s specimens grown in the coastal area of northwestern Sicily (Abruscato et al. 2023). In the same publication, we also reported the appearance of a dose-dependent cytotoxic effect of both preparations on liver cancer cells, with the modulation of apoptosis, autophagy, cellular redox and mitochondrial states otherwise implicated in the death-inducing mechanisms. Here, we sought to expand the knowledge on the biomedical implementations of GLE and RE by investigating whether sublethal concentrations of the preparations can exert glucose-lowering effects in vitro on the HepG2 liver cancer cell line. The liver is the main organ where glucose metabolism and storage occur and the main target for the molecular changes in glucose internalization and utilization. Therefore, since HepG2 cells express several differentiated liver functions, including glycogen storage and insulin signaling, and are currently considered a valuable tool for identifying drug candidates targeting the insulin/PI3 K/Akt pathway in the liver, they were chosen as a model system for study (Donato et al. 2015; Yudhani et al. 2023). Using a combination of cytochemical, flow cytometric, PCR and protein blot techniques, the aim of our study was to examine the impact of GLE and RE on glucose internalization and utilization. We also studied the expression of two glucose transporters, the insulin-insensitive GLUT-2 and GLUT-4 whose exposure at cell surface is controlled by insulin (Karim et al. 2012). The investigation also included the evaluation of the expression level of the *GLUT-2*-related transcription factor HNF1α (Kim et al. 2017), and the analysis of the activation states of members of the insulin-dependent signaling pathway involved in the translocation of GLUT-4 from intracellular storage compartments to the plasmalemma, i.e., insulin receptor substrate-1 (IRS-1), protein kinase B (AKT) and PKCζ (Hah 2000; Liu et al. 2007; Ramachandran and Saravanan 2015; Mokashi et al. 2017). In fact, in hepatic cells the GLUT-4 transporter, which is present in reduced amounts, serves as a high-affinity carrier that promotes glucose uptake upon insulin binding to its receptor, consequently activating the IRS-1 signaling cascade. This mechanism may induce a divergence by phosphorylating two specific downstream kinase effectors, namely AKT and/or protein kinase C ζ (PKCζ), thereby initiating the translocation of GLUT-4. Of note, the phosphorylation of IRS-1 at its serine and threonine locations inhibits the signaling cascade, thus preventing the accumulation of the transporter on the cell surface in response to insulin.

## Materials and methods

### Preparation of GLE and RE

The GLE and RE used for the present study were prepared as previously reported by Abruscato et al. (2023) and Punginelli et al. (2023). Briefly, the powdered rhizomes and green leaves of *P. oceanica* samples collected during the summer season in the Gulf of Palermo (Sicily, Italy) were individually subjected to extraction in the presence of acetic acid and protease inhibitors. After homogenization and sonication, the filtered GLE and RE were lyophilized and stored at − 20 °C until use. The HPLC/MS analysis of the polyphenol content and the MS-based proteomic study of the two preparations have already been published by Abruscato et al. (2023).

### Cell culture and treatment

HepG2 liver cancer cells (RRID: CVCL_0027) taken from laboratory stocks were grown in high glucose–DMEM medium (D642; Sigma, St. Louis, MO/USA) supplemented with 10% fetal calf serum (F4135; Sigma) and an antibiotic/antimycotic solution (A5955; Sigma) in a humidified incubator at 37 °C and an atmosphere of 5% CO_2_. In view of the previous results of viability tests (Abruscato et al. 2023), the maximum sublethal concentrations of GLE and RE corresponding to 60 and 2.2 μg/mL, respectively, were selected for subsequent experiments.

### Assessment of glycogen storage

HepG2 cells were plated at a density of 88,000 cells/well in 6-well plates and, once subconfluent, were treated with either GLE or RE, with or without the addition of 10^−7^ M insulin (Santa Cruz Biotechnology, Heidelberg, D), or 10^−7^ M insulin alone for 24 h. Untreated cells were used as controls. Glycogen storage was assessed by the PAS reaction as reported by Donato et al. (2015). Briefly, the cell cultures were fixed with 4% paraformaldehyde solution in PBS (Santa Cruz Biotechnology), treated with 0.5% periodic acid solution (Santa Cruz Biotechnology) and stained with Schiff’s reagent (Santa Cruz Biotechnology). Pink intracellular glycogen deposits were visualized under the light microscope and photographed, and the mean density value of triplicate assays for each preparation was measured using the ImageJ software.

### Measurement of glucose consumption

Glucose consumption was assessed by measuring its concentration in the medium of control and treated cells (Abruscato et al. 2024). Briefly, HepG2 cells were plated at a concentration of 40,000 cells/well in 24-well plates and, once subconfluent, grown in control conditions or treated with either GLE or RE, with or without the addition of 10^−7^ M insulin, or 10^−7^ M insulin alone for 24 h. After incubation, the medium was collected, centrifuged, diluted in half with sterile distilled water and the glucose concentration was measured in triplicate with a glucometer (Glucomen Areo 2 k, Menarini Diagnostics, Firenze, Italy) and single-use test strips (Glucomen Areo Sensor, Menarini Diagnostics). All media underwent testing for their baseline glucose concentrations before being administered to the cells, to ensure the absence of interference with the results of the experiment.

### Flow cytometry

Flow cytometric analyses were performed in a FACSCanto flow cytometer (BD Biosciences, Franklin Lakes, NJ, USA) evaluating 10,000 single-cell events. The obtained fcs files were analyzed with the online tool Floreada (https://floreada.io; accessed on 6 March 2024). Gating in the FSC vs. SSC plot was performed for each analysis to exclude cell debris which showed low FSC values.

Glucose uptake was determined using the fluorescent analog of d-glucose, 2-[N-(7-nitrobenz-2-oxa-1,3-diazol-4-yl) amino]−2-deoxy-d-glucose (2-NBDG; Peptide Institute, Osaka, Japan) (Abruscato et al. 2024). HepG2, plated at a concentration of 88,000 cells/well in 6-well plates, once subconfluent were exposed to either GLE, RE or 10^−7^ M insulin for 24 h. Then, after removing the medium, the cells were treated with 2-NBDG in Ca^++^/Mg^++^-containing PBS for 1 h, detached by trypsinization and immediately subjected to flow cytometric measurement of 2-NBDG uptake in the fluorescein isothiocyanate (FITC) channel. Propidium iodide (PI) staining was performed in parallel to determine the effect of the treatment on cell death. Untreated cells were analyzed as controls.

For immunostaining and quantitative evaluation of plasmalemma-exposed GLUT-4 transporter HepG2 cells were seeded at a concentration of 88,000 cells/well in 6-well plates and, once subconfluent, treatments were applied for 24 h. Untreated cells were used as controls. Then, the cells were detached by trypsinization, washed with ice-cold PBS, and incubated for 20 min in the cold with rabbit anti-GLUT-4 primary antibody (bs-0384R-TR, Bioss), diluted to a working concentration of 1:100 in PBS containing 3% BSA. An isotype control was included in the assay. Incubation with the FITC-conjugated secondary antibody (AP132 F, Sigma, RRID: AB_92490; working dilution 1:80) was performed for an additional 20 min, and then, the cell preparations were exhaustively washed and promptly subjected to flow cytometry analysis (Abruscato et al. 2024).

### RNA isolation, reverse transcription and RT-qPCR

mRNA expression analysis was performed by RT-qPCR. Basically, total RNA from control and treated cells was extracted using the PureLink RNA Mini kit (ThermoFisher). The PureLink DNase set (ThermoFisher) was used following the manufacturer’s instructions for on-column DNase treatment. Five hundred ng of each RNA preparation were used to synthesize the cDNA with the RevertUP™ II Reverse transcriptase kit (Biotechrabbit, Berlin, Germany) and random hexamers according to the manufacturer’s instructions.

Utilizing the Applied Biosystems 7500 Real-time PCR system (Applied Biosystems, Waltham, MA/USA), the RT-qPCR analysis was performed employing the SYBR Green qPCR Master Mix (MedChem Express, Monmouth Junction, NJ/USA) along with the specific primer sets indicated in Table 1 (Abruscato et al. 2023). The specificity of the amplification was assessed through the application of RT-qPCR melting analysis. The samples were quantified using the 2^−ΔΔCt^ methodology, with transcript levels standardized to *ACTB* to compensate for variations in the quantity of RNA input. The analysis of relative expression was performed by computing the ratio of the normalized values of the target gene in treated samples to those obtained from control samples.

**Table 1:** Primers used for PCR amplification

Gene (primer)	Sequence (5′ → 3′)
*GLUT2* (sense)	GATGAACTGCCCACAATCTC
*GLUT2* (antisense)	CTGATGAAAAGTGCCAAGTG
*GLUT4* (sense)	GTTAATCGGCATTCTGATCG
*GLUT4* (antisense)	GTGAAGACTGTGTTGACCAC
*AKT2* (sense)	GCTAGGTGACAGCGTACCAC
*AKT2* (antisense)	GGCCTCTCGGTCTTCATCAG
*IRS1* (sense)	TATCTGCATGGGTGGCAAGG
*IRS1* (antisense)	GGGTAGGCAGGCATCATCTC
*HNF1A* (sense)	GAATGCATCCAGAGAGGGGT
*HNF1A* (antisense)	GTGGACCTTACTGGGGGAGA
*ACTB* (sense)	GGAAGGTGGACAGCGAGGC
*ACTB* (antisense)	GTGACGTGGACATCCGCAAA

### Western blot analysis

The amounts of GLUT-1, GLUT-4, HNF1α, AKT, phospho-AKT (pAKT), IRS-1, phospho-IRS1 (pIRS-1) (Ser307), and phospho-PKCζ (pPKCζ) proteins in control and treated cell samples were assessed by Western blotting as previously reported (Abruscato et al. 2023). Briefly, total protein samples were extracted from control and treated cells in lysis buffer (7 M Urea, 2% CHAPS, and 10 mM DTT, all from Sigma) containing a cocktail of protease inhibitors (Promega, Madison, WI/USA), separated by 13% SDS-PAGE, and transferred to nitrocellulose membranes. The membranes were probed with one of the following rabbit primary antibodies at 4 °C overnight: anti-GLUT2 (bs-10379R-TR, Bioss, Boston, MA/USA; working dilution 1:500), anti-GLUT4 (bs-0384R-TR, Bioss; working dilution 1:500), anti-HNF1α (PAG775Hu01, Cloud-Clone Corp., Katy, TX/USA; working dilution 1:1000), anti-AKT (9272, Cell Signaling Technology, Danvers, MA/USA; working dilution 1:750), anti-pAKT (sc-7985-R, Santa Cruz Biotechnology, working dilution 1:500), anti-IRS1 (NB-22–3767, NeoBiotech, Nanterre, F, working dilution 1:1000), anti-pIRS1 Ser307 (NB-22–0306, NeoBiotech, working dilution 1:1000), anti-pPKCζ Thr410 (MA5-46896, Invitrogen, Waltham MA/USA, working dilution 1:1000) and, as an internal control, anti-actin (Ab8227, Abcam, Cambridge, UK; working dilution 1:1000). After reaction with the peroxidase-conjugated secondary antibody (Ab6721, Abcam, RRID: AB_955447; working dilution 1:3000) at room temperature for 1 h, the bands were visualized by an enhanced chemiluminescence system (Versadoc MP Imaging System, Bio-Rad, Hercules, CA/USA) using the SuperSignal West Pico Plus substrate (ThermoFisher). Each signal intensity was quantified using the ImageJ software with normalization to that of the actin band.

### Statistics

Data are presented as mean ± standard error of the mean (s.e.m.) of triplicate experiments. One-way variance analysis (ANOVA) with Holm-Sidak post-hoc test and Shapiro–Wilk normality tests were performed using the SigmaPlot 11.0 software (SYSTAT, San Jose, CA, USA). For PCR and Western blot experiments, the data were analyzed by an unpaired two-tailed Student’s *t*-test using the GraphPad Prism 9 software (GraphPad, San Diego, CA, USA).

## Results

### GLE stimulates glycogen accumulation in HepG2 cells

**Fig. 1:**
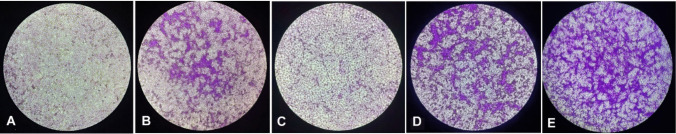
GLE upregulates glucose storage in HepG2 cells. Representative PAS staining images showing glycogen accumulation in HepG2 cells grown under control conditions (**A**) and in the presence of 10^−7^ M insulin (**B**), 2.2 μg RE/mL (**C**), 60 μg GLE/mL (**D**), and 60 μg GLE/mL + 10.^−7^ M insulin (**E**). Stained preparations of control and treated cells were examined under the light microscope, photographed, and processed for quantification of the mean density values using the ImageJ software, as presented in Table 2. Microscopic magnification =  × 10

In the first series of tests aimed at evaluating the effect of *P. oceanica* extracts on glucose metabolism in HepG2 cells, PAS staining was performed to detect glycogen storage under the different culture conditions. As shown in the panel in Fig. 1, the PAS assay showed that, as expected, cells cultured for 24 h in the presence of insulin showed increased glycogen storage compared to controls. Interestingly, the results obtained from exposure to GLE were in opposition to those derived from RE treatment. Indeed, unlike RE which exhibited no significant effect, glycogen accumulation in cells cultured in the presence of GLE was higher than in cells treated with insulin. It is noteworthy that co-treatment with GLE plus insulin did not result in a significant additional upregulation of intracellular glycogen storage. Table 2 reports the quantitative values of the mean staining density of each preparation. The staining in the presence of RE and insulin co-treatment was comparable to that with insulin alone (not shown).

**Table 2:** Quantitation of the mean density values of PAS staining (average ± s.e.m of triplicate assays)

A	B	C	D	E
82.1 ± 5	104.4 ± 6.3	81.3 ± 4.4	128.4 ± 7	133 ± 6.7

### GLE increases glucose consumption and glucose uptake by HepG2 cells

**Fig. 2:**
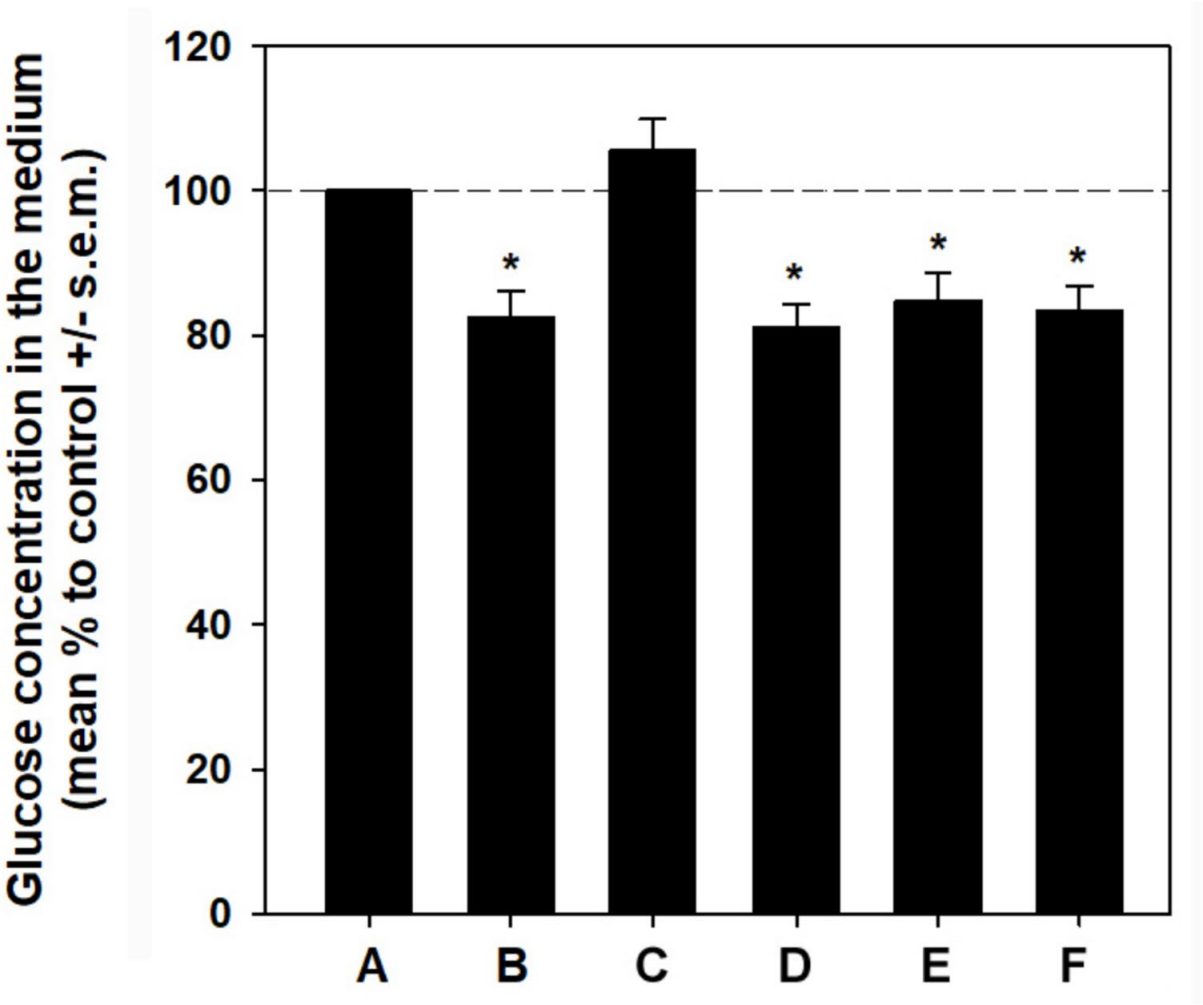
GLE increases glucose consumption by HepG2 cells. Bar graph showing the percentage concentration of glucose in the culture medium of HepG2 cells grown in the presence of 10^−7^ M insulin (**B**), 2.2 μg RE/mL (**C**), 60 μg GLE/mL (**D**), 60 μg GLE/mL + 10^−7^ M insulin (**E**), and 2.2 μg RE/mL + 10^−7^ M insulin (**F**) compared to control (**A**). Each medium was collected, centrifuged, and diluted, and glucose concentration determined in triplicate using a glucometer and single-use test strips. Each bar shows the mean ± s.e.m. of the measurements reported in Table S1. *p < 0.05 vs. control. Normality test vs. control passed

It is known that the quantification capabilities of PAS staining may be limited for the fact that glycogen granules must reach a diameter of at least 50 nm to be observed using standard light microscopy, coupled with the possibility of encountering technical false positives (Tabatabaei Shabiei et al. 2014). Therefore, the potential glucose-lowering activity of GLE and RE was further evaluated by glucose consumption and glucose uptake assays. A preliminary evaluation of the glucose content in the control and supplemented media demonstrated a lack of statistically significant differences, implying that their intrinsic composition did not lead to any potential interferences in the assay (fig. S1). As shown in Fig. 2, in agreement with the previous results the glucose concentration in the culture medium of HepG2 cells exposed to GLE for 24 h decreased to 82.5 ± 5% of the control, a value similar to that obtained in the presence of insulin (81.1 ± 5.6%). In contrast, no statistically significant difference was found upon RE treatment. Co-incubation with GLE and insulin did not exert a statistically significant synergistic effect, maintaining a similar degree of reduction in glucose level in the culture medium (84.7 ± 3.8%) as that of the individual treatments. Furthermore, co-incubation with RE and insulin resulted in glucose consumption comparable to that of cells exposed to insulin alone. Therefore, due to the lack of significance of the glucose measurement and the PAS assay results, both co-incubation conditions were not included in subsequent experiments.

**Fig. 3:**
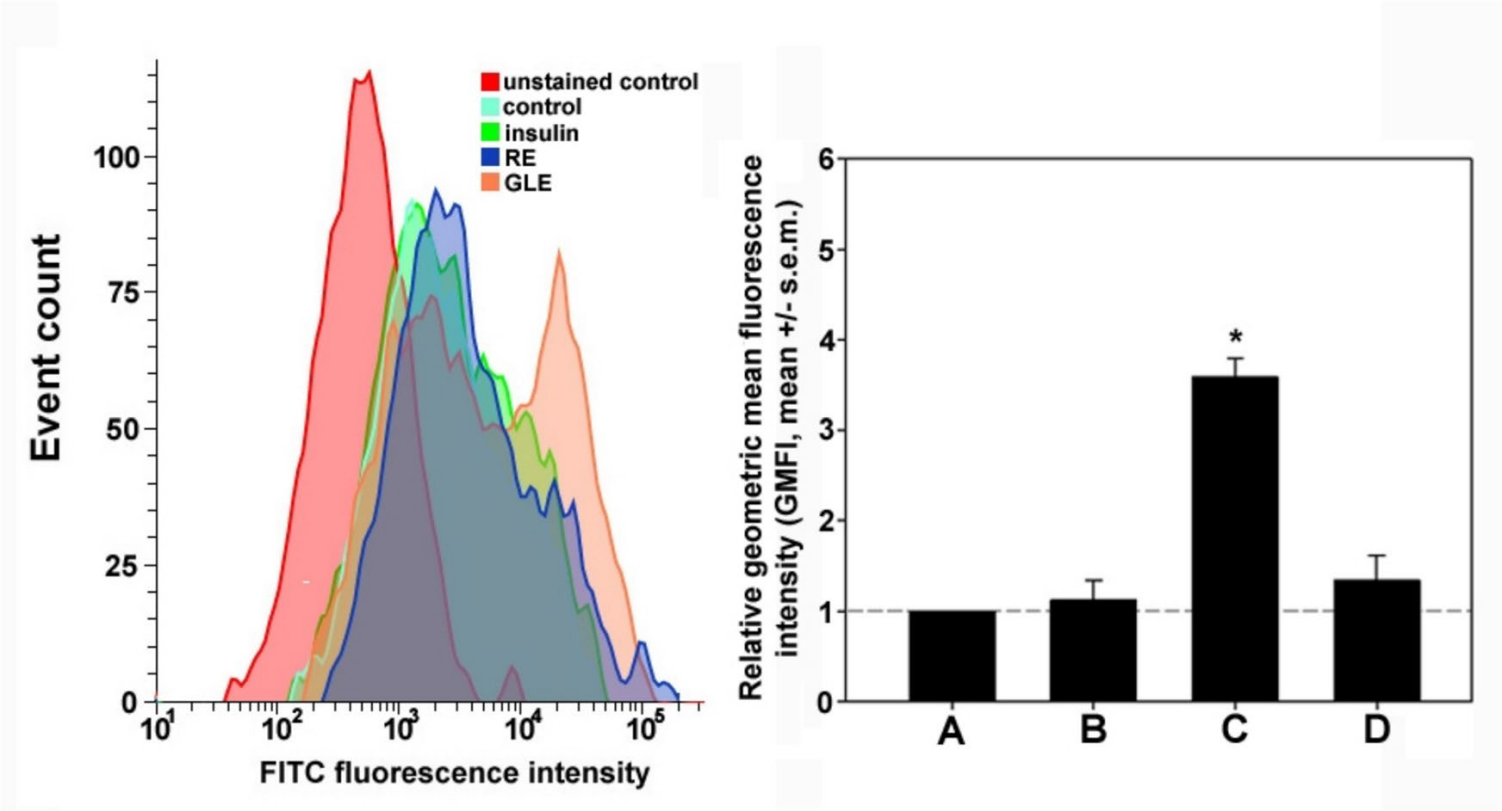
GLE upregulates short-term glucose uptake by HepG2 cells. (Left) Representative flow cytometric profiles of 2-NBDG uptake by HepG2 cells cultured for 1 h under control conditions or treated with 10^−7^ M insulin, 2.2 μg RE/mL or 60 μg GLE/mL. Fully processed preparations without 2-NBDG were analyzed in parallel for the control of background derived from autofluorescence (unstained control). (Right) Bar graph showing the relative geometric mean fluorescence intensity (GMFI) of control (**A**), insulin- (**B**), GLE- (**C**), or RE-treated (**D**) HepG2 cells. Control and treated cells were exposed to 2-NBDG for 1 h, trypsinized, and immediately subjected to flow cytometry analysis. All the results were analyzed for the GMFI of each condition and the GMFI of either treated cell population was divided by the GMFI of the controls to normalize the data. Error bars indicate the s.e.m. of three independent measurements. * p < 0.05 vs. control. Normality test vs. control passed

We then evaluated the effect of GLE and RE on short-term glucose uptake by HepG2 cells using the fluorescently labeled non-metabolizable glucose analog 2-NBDG. The results obtained, shown in Fig. 3, demonstrated that after 1 h of treatment only GLE induced a significant and immediate response leading to the stimulation of 2-NBDG internalization by approximately 2.6 ± 0.03-fold compared to the control. Conversely, no significant increase in short-term uptake was seen after exposure to RE (+ 0.3 ± 0.3-fold) or insulin alone (+ 0.1 ± 0.2-fold). Based on these and previous results, only GLE-treated cells were subjected to further studies on the molecular aspects of the increased glucose absorption.

### GLE induces the upregulation of IRS-1/AKT/GLUT-4 pathway and of GLUT-4 exposure on HepG2 cell surface, while downregulating GLUT-2

In a third set of experiments, we examined whether exposure of HepG2 cells to GLE could modify the gene expression and/or protein accumulation levels of the respectively insulin-insensitive and insulin-stimulated GLUT-2 and GLUT-4 transporters. The same study was also performed on other regulators, such as the *GLUT2* transcription factor HNF1α (Párrizas et al. 2001; Kim et al. 2017), IRS-1 and AKT with their activated forms and pPKCζ, members of a signaling pathway responsible for the translocation of GLUT-4 from intracellular storage compartments to the plasmalemma (Hah 2000; Świderska et al. 2018; Zhang et al. 2019). In parallel, preparations obtained after cell exposure to 10^−7^ M insulin were tested.

**Fig. 4:**
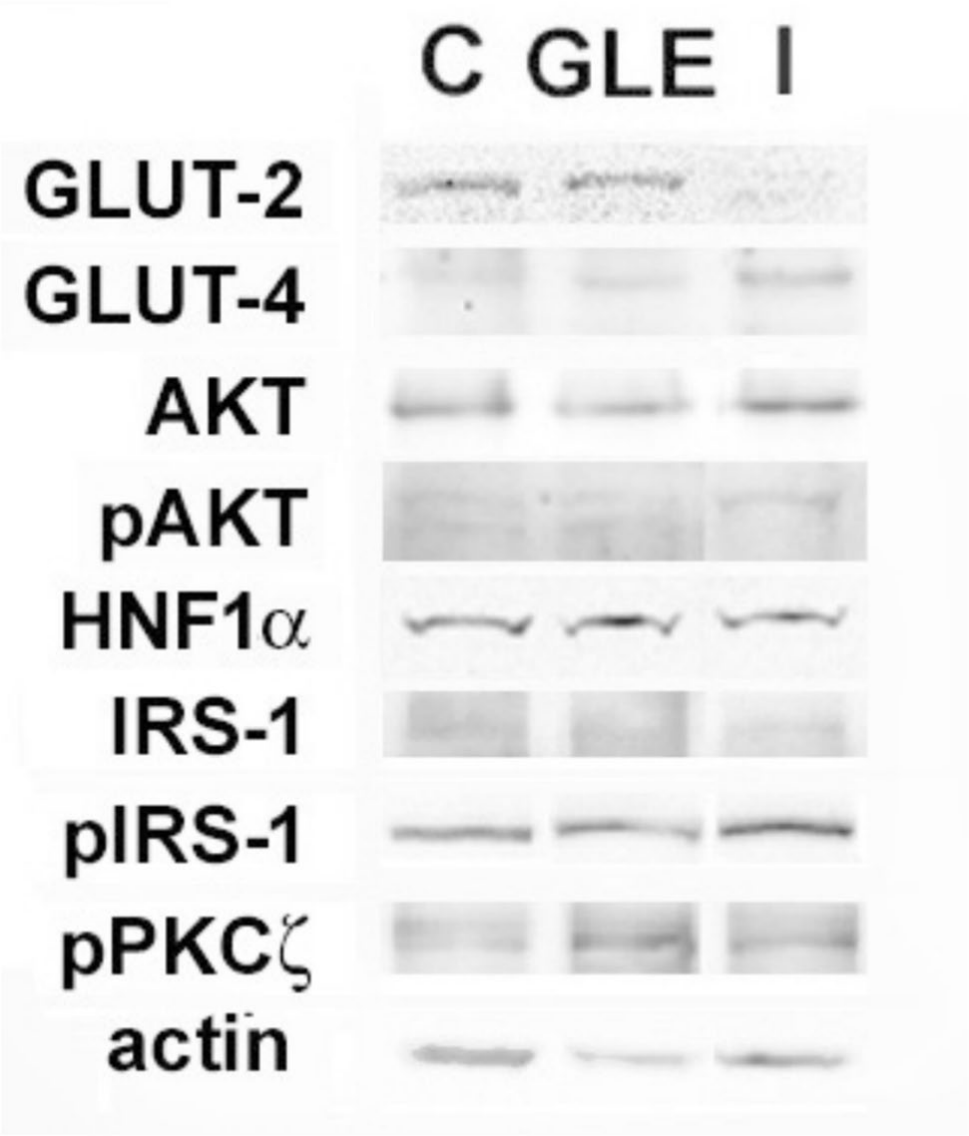
GLE modulates protein expression in HepG2 cells. Representative results of Western blot analysis for the accumulation of glucose transporters and regulators in HepG2 cells cultured for 24 h under control condition or in the presence of 60 μg GLE/mL (GLE) or 10^−7^ M insulin (I)

The results of RT-qPCR experiments are reported in Table 3. Figure 4 shows the protein data panel obtained by Western blot, and the densitometric analysis performed with the ImageJ software is reported in Table 4.

**Table 3:** Relative quantification of RT-qPCR (treated/control ratio expressed as mean ± s.e.m. of triplicate experiments). * *p* < 0.05 vs. control. Normality test vs. control passed

	GLE	I
*GLUT2*	0.99 ± 0.05	1.7 ± 0.05*
*GLUT4*	0.72 ± 0.01*	1.56 ± 0.06*
*IRS1*	1.11 ± 0.01	0.48 ± 0.02*
*AKT2*	0.92 ± 0.02	0.92 ± 0.07
*HNF1 A*	0.43 ± 0.03*	0.5 ± 0.02*

**Table 4:** Relative quantification of proteins (treated/control ratio expressed as mean ± s.e.m. of triplicate experiments) obtained by band densitometry and normalization to actin. * *p* < 0.05 vs. control. Normality test vs. control passed

	GLE	I
GLUT-2	0.6 ± 0.03*	0.31 ± 0.02*
GLUT-4	4.4 ± 0.09*	2.08 ± 0.04*
HNF1α	1.06 ± 0.02	1.87 ± 0.02*
pAKT/AKT	4.2 ± 0.09*	2.73 ± 0.03*
pIRS-1 (Ser307)/IRS-1	0.76 ± 0.06*	2.35 ± 0.03*
pPKCζ	2.23 ± 0.07*	1.06 ± 0.03

Data analysis demonstrates that GLE treatment for 24 h led to a downregulation of *GLUT4* and *HNF1 A*, while *GLUT2*, *IRS1*, and *AKT2* remained largely unaffected. The parallel assessment of protein expression revealed that (i) GLE induced an upregulation of GLUT-4 while decreasing GLUT-2 levels, (ii) no significant changes were observed in HNF1α levels, and, interestingly, (iii) GLE triggered an increase in the activation of AKT and PKCζ as well as a marked downregulation of the inhibitory phosphorylated form of IRS-1. Analysis of mRNA from cells exposed to insulin for 24 h indicated (i) a slight upregulation of GLUT-2 and GLUT-4 transcripts, (ii) a decrease in IRS-1 and HNF1a mRNA levels, and (iii) no significant changes in *AKT2* expression. The analysis conducted on protein samples showed that (i) the upregulation of GLUT-4 was less pronounced than in response to GLE, (ii) GLUT-2 exhibited a more prominent downregulation relative to GLE, even with an apparent increase in HNF1α, and, interestingly, (iii) a pronounced inhibitory phosphorylation of IRS-1 occurred, along with a reduced activation of AKT and no change in pPKCζ amount. The whole images of the electrophoretic gels and blots are shown in Fig. S2. The apparent discrepancy between the decrease in the amount of transcripts and the increase of the corresponding protein levels, as observed with *GLUT4* in GLE-treated cells and *HNF1 A* in insulin-treated cells, may result from various factors, including the efficiency of translation and the occurrence of post-transcriptional regulation or modifications that influence protein stability or functionality, which will be the object of future study in our experimental system.

**Fig. 5:**
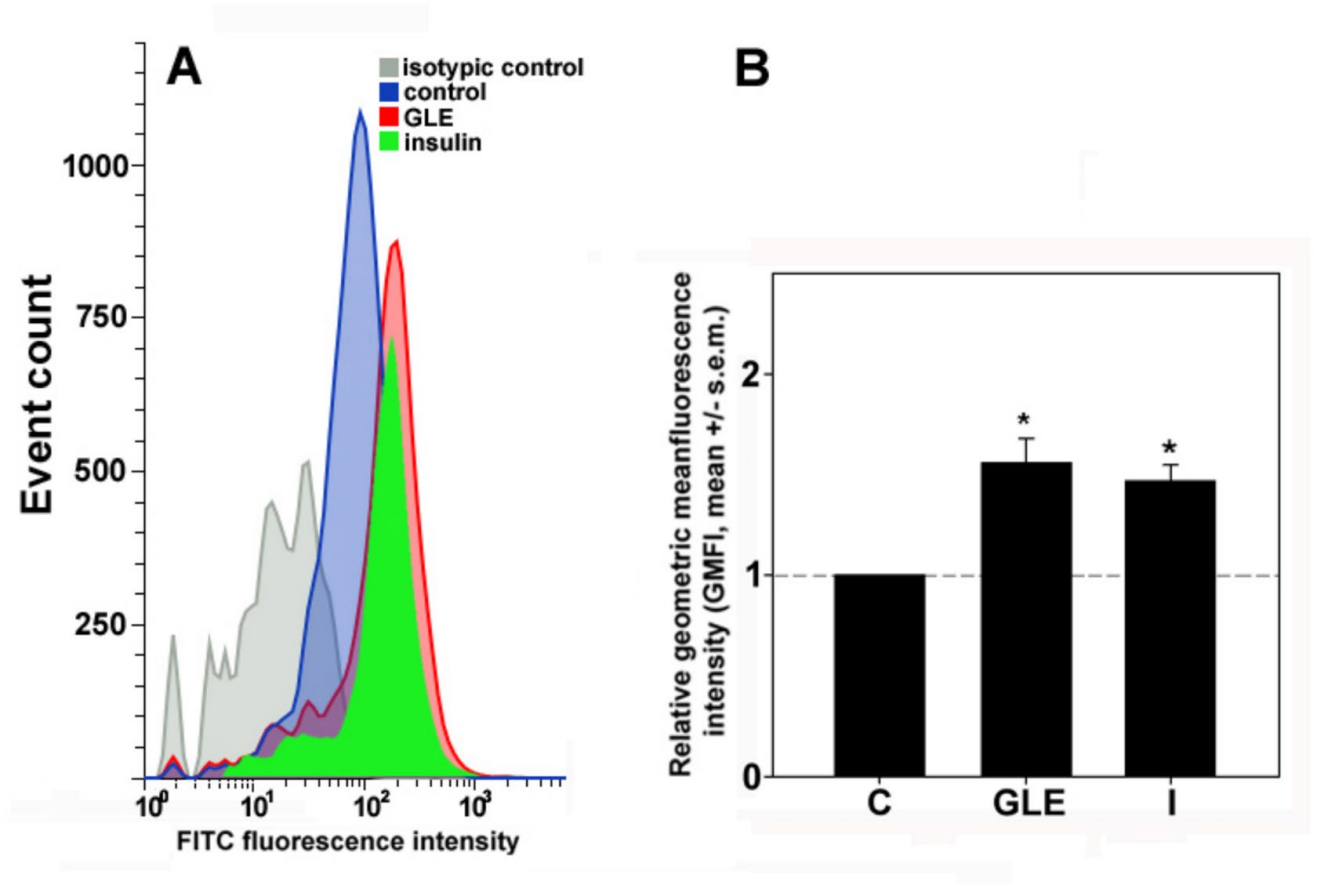
GLE increases GLUT-4 exposure at the plasma membrane of HepG2 cells. **A** Representative flow cytometric profiles showing the amount of immunostained GLUT-4 transporter exposed on the surface of HepG2 cells cultured for 24 h under control conditions or treated with either 60 μg GLE/mL or 10^−7^ M insulin. **B** Bar graph showing the relative geometric mean fluorescence intensity (GMFI) of GLUT-4 in cells exposed to either GLE or insulin (I), normalized to the control (C). Cumulative data from flow cytometry experiments were analyzed for the GMFI of each condition and the GMFI of the treated cell population was divided by the GMFI of the controls to normalize the data. Error bars indicate the s.e.m. of three independent measurements. *p < 0.05 vs. control. Normality test vs. control passed

In view of the previous results, to examine whether the upregulation of GLUT-4 and the activation of IRS1, AKT, and PKCζ after GLE treatment could lead to enhanced transporter translocation to the plasma membrane, intact HepG2 cells were immunostained for the surface GLUT-4 content and analyzed by flow cytometry. Insulin-treated cells were analyzed in parallel. As shown in Fig. 5, both the extract and insulin, although the latter to a lesser degree, were found to significantly induce GLUT-4 exposure (about + 52 and + 47%, respectively) at the cell membrane.

## Discussion

Several potential applications of fresh and dead *P. oceanica*’s waste have been recently proposed, such as the production of fibers for biocomposites and animal feed, biochar, compost and bioethanol, and the extraction of compounds for biomedical use (Cocozza et al. 2011; Ferrández-Gómez et al. 2023; Souii et al. 2023). The latter application cannot ignore study strategies aimed at a comprehensive understanding of their mechanisms of action, including pre-clinical research at the cellular and molecular levels. The current study was designed to examine whether the water-soluble extracts from two different anatomical parts, i.e., the rhizomes and green leaves, of the seagrass *P. oceanica* grown along the northern coast of Sicily can have a positive effect on glucose metabolism in cultured liver cells. The data obtained revealed the promising efficacy of GLE as another potential anti-diabetic agent, while RE showed no effect.

The glucose metabolic dysfunction that occurs in diabetes mellitus sees the nodal involvement of the GLUT transporters. In hepatocytes, in particular, the glucose flux is mediated primarily by GLUT2, the insulin-independent transporter, which is responsible for glucose uptake but, in some cases, also for its output, thereby controlling the balance between intracellular and extracellular glucose concentrations (Thorens 2015). Interestingly, Yonamine et al. (2017) have reported that restoration of glycemic homeostasis by resveratrol in diabetic mice involved a decrease in *GLUT2* expression in the liver, probably due to reduced hepatic glucose outflow. On the other hand, *GLUT4* is characterized by its responsiveness to insulin signaling that causes the translocation of GLUT4-containing vesicles to the membrane via the insulin-stimulated IRS1/PI3K/AKT and PKCζ pathway (Hah 2000; Kouznetsova et al. 2017). Thus, a disruption in the levels of GLUT expression as well as a reduction in the recruitment of GLUT-4 from cytoplasmic vesicles and its positioning on the cell surface are molecular alterations that contribute to impaired glycemic control in diabetes mellitus. The data obtained show that, as has been reported for other natural products (e.g., Zhang et al. 2015; Chen et al. 2017; Kim et al. 2017; Mokashi et al. 2017), GLE caused an enhanced activation of IRS-1, AKT and PKCζ, and an upregulation of the translation levels of GLUT-4. On the other hand, in the present study, we did not find a significant change in HNF1α protein while we observed a decrease in GLUT-2 levels in GLE-treated cells. Concerning insulin-treated cells, the expression pattern of the two transporters mirrored that of GLE, but the increase in GLUT-4 was less evident; noteworthy, the IRS-1 signaling pathway appeared restricted. This situation may correlate with the emergence of insulin resistance, a condition that has been reported following a 24-h treatment of HepG2 cells with the hormone at the concentration used in this analysis (Huang et al. 2015). An alternative hypothesis is that the downregulation of GLUT-2 may be linked to a reduction in glucose efflux by HepG2 liver cells, as indicated by Yonamine et al. (2017), although further experiments are needed to confirm this correlation. Interestingly, insulin and, to a greater extent, GLE have been shown to stimulate the translocation of GLUT-4 to the cell membrane. This is consistent with the increased glucose consumption and uptake observed with both treatments, likely counteracting cellular depletion of GLUT-2.

The polyphenolic and protein compositions of the GLE used in the present experiments have been studied and reported by Abruscato et al. (2023). Previous study by Leri et al. (2018) indicates that the polyphenols from *P. oceanica* can modulate insulin signaling pathways at the cellular level, resulting in greater insulin efficacy and more effective glycemic control. The number and nature of the polyphenolic compounds found in the two different *P. oceanica* matrices here used were consistent with those reported in the literature (Farid et al. 2018; Ammar et al. 2021; Messina et al. 2021). The differences observed may be related to the extraction method used and the amounts extracted. The extracellular glucose-lowering and GLUT-4 upregulating effects in HepG2 cells might be ascribed to the specific polyphenols found in GLE, which are absent in RE. The predominant component of the preparation is caffeic acid methyl ester, a phenolic compound belonging to the class of hydroxycinnamic acids, known to play a significant role in increasing insulin sensitivity and regulating insulin signaling pathway in peripheral tissues. In GLE, trace amounts of ellagic acid, kaempferol, *p*-coumaric acid and procyanidin B2 are also present. Noteworthy, caffeic acid methyl ester and the other minor constituents found in GLE have been shown to significantly stimulate the uptake of glucose in hepatic tissues or cultured liver cells. This primarily happened through the upregulation of GLUT-4 expression and its translocation activated by insulin- and/or 5’AMP-activated protein kinase (AMPK)-dependent pathways (Chen et al. 2010; Ouchfoun et al. 2016; Eid et al. 2017; Liu and Li 2021; ALTamimi et al. 2022).

In relation to the protein component, it is significant to consider that the proteomic study by Abruscato et al. (2023) did not reveal the presence of insulin analogs in GLE.

In this work, we have produced in vitro data supporting the glucose-lowering potential of GLE from *P. oceanica* in an experimental model of cultured liver cells. The panel of cellular and molecular analyses performed demonstrated improved glucose uptake and consumption, and a molecular reprogramming that included the upregulation of (i) IRS-1, AKT, and PKCζ activation levels, (ii) GLUT-4 translation levels, and (iii) GLUT-4 exposure at the cell surface. Of note, the comparative study performed with GLE and RE clearly shows that the phytocomplexes extracted from the two different anatomical parts of the seagrass, that have different components, do not share the same bioactive potential. Thus, it is strongly recommended that future indications of the health benefits of *P. oceanica*’s extracts should explicitly mention the source of the preparation. From the point of view of an industrial application, the extraction of this biological matrix is simple and inexpensive, and also without methodological problems. Although the component(s) of GLE to which the observed effects can be attributed has (have) not been isolated, phytochemical analysis nonetheless suggests the presence of polyphenols that apparently may be involved in facilitating glucose consumption and uptake through the implementation of GLUT-4 activity. The “active principle(s)” of the phytocomplex is/are resistant to lyophilization, resuspension, and freeze–thaw cycles, which are the procedures used in the preparation of GLE before bioassays. We cannot rule out the contribution of other water-soluble non-polyphenolic components, as well as the occurrence of synergistic activities between the substances present.

Overall, the action related to glucose metabolism detected here adds up to the previously demonstrated anticancer effect of the extract (Abruscato et al. 2023), thus reinforcing interest in the biomedical implementation of this seagrass-derived preparation. Products of marine origin have great potential to be used in the food industry as direct and indirect additives, barrier coatings for multilayer packaging or edible coatings applied directly to food (Shahidi and Ambigaipalan 2015; Kumar et al. 2022). Given the great need to develop alternative treatment options for diabetes mellitus, our results support the concept that the aqueous extract of green leaves of *P. oceanica* or its derivatives are of interest for the development of new agents for the treatment of hyperglycemia and beneficial supplements for the formulation of functional foods and food packaging materials.

## Supplementary Information

Below is the link to the electronic supplementary material.

Supplementary file1 (PDF 384 KB)

## Data Availability

All data generated or analyzed during this study are included in this published article and its supplementary information file.
