# Supplementary material for: Glucose consumption and uptake in HepG2 cells is improved by aqueous extracts from leaves, but not rhizomes, of Posidonia oceanica (L.) Delile via GLUT-4 upregulation
Source: Protoplasma. 2025 May 24;262(6):1483–93. doi: 10.1007/s00709-025-02076-8 (PMC12535535; doi:10.1007/s00709-025-02076-8)
Supplement: Supplementary file 1 — Supplementary file1 (PDF 384 KB) [file 709_2025_2076_MOESM1_ESM.pdf]

Glucose consumption and uptake in HepG2 cells is improved by aqueous extracts from leaves, but not rhizomes, of *Posidonia oceanica* (L.) Delile *via* GLUT-4 up-regulation

Giulia Abruscato[a], Roberta Tarantino[a], Manuela Mauro[a], Roberto Chiarelli[a], Aiti Vizzini[a,b], Vincenzo Arizza[a,b], Mirella Vazzana[a,b] and Claudio Luparello[a,b]*

Supplemental information

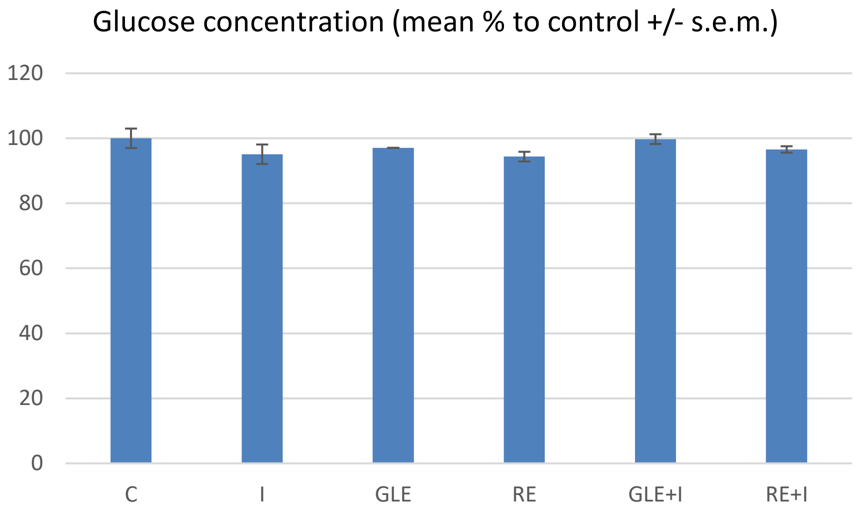

Figure S1: Bar graph showing the percentage concentration of glucose in the culture medium supplemented with 10^{-7} M insulin (I), 60 µg GLE/mL (GLE), 2.2 µg RE/mL (RE), 60 µg GLE/mL + 10^{-7} M insulin (GLE + I) and 2.2 µg RE/mL + 10^{-7} M insulin (RE + I) compared to control (C). No statistically significant difference can be observed (p = 0,090).

Table S1. Glucose content (mg/dl) in the different culture media evaluated in two triplicate assays

**Experiment nr. 1**					
C	I	GLE	RE	GLE + I	RE + I
224	185	184	223	190	188
211	187	196	219	191	188
210	188	184	223	190	190
**Experiment nr. 2**					
C	I	GLE	RE	GLE + I	RE + I
193	147	150	209	156	155
189	144	150	210	157	151
200	147	151	209	158	153

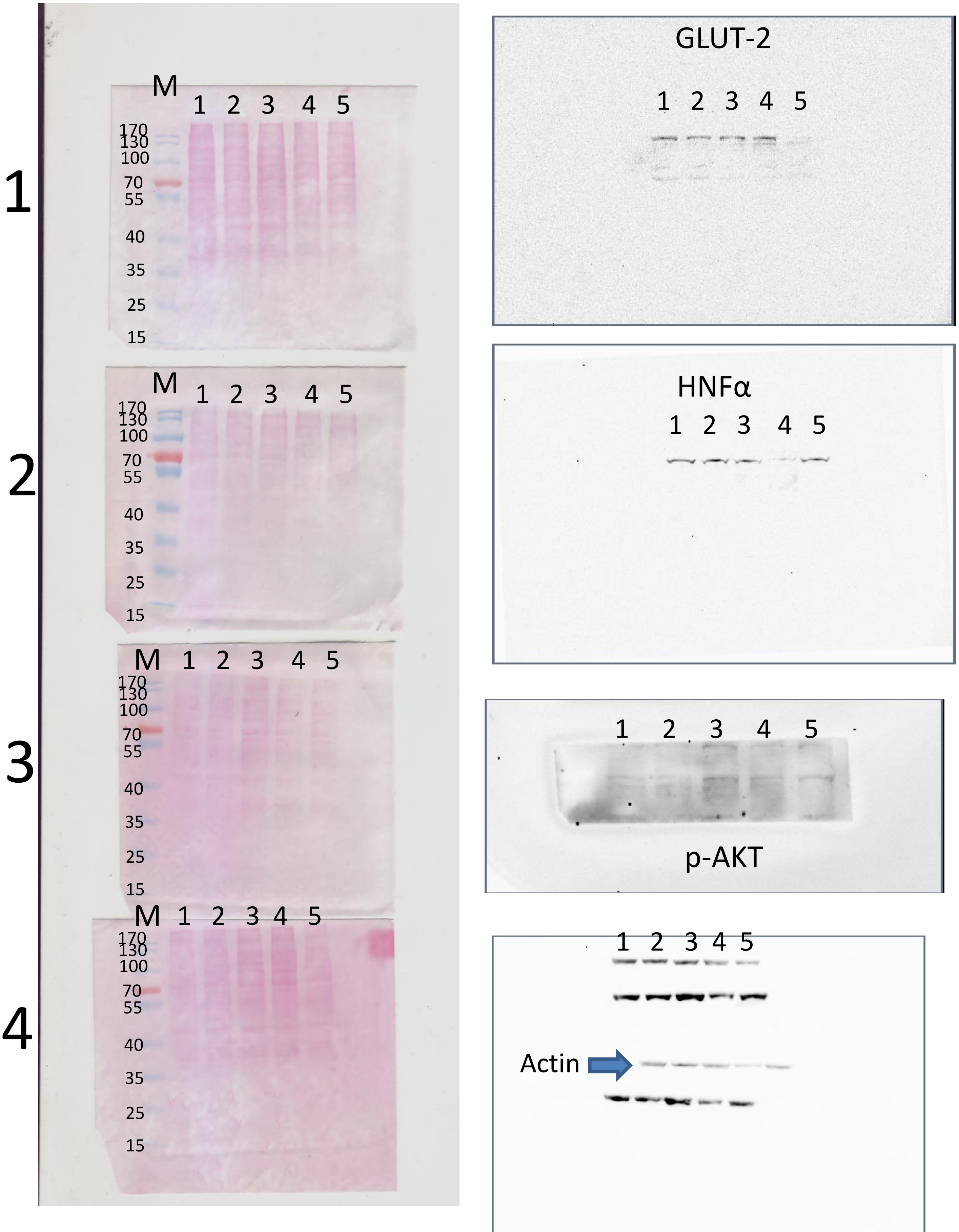

1
M 1 2 3 4 5
170 130 100 70 55 40 35 25 15
GLUT-2
1 2 3 4 5
2
M 1 2 3 4 5
170 130 100 70 55 40 35 25 15
HNFα
1 2 3 4 5
3
M 1 2 3 4 5
170 130 100 70 55 40 35 25 15
1 2 3 4 5
p-AKT
4
M 1 2 3 4 5
170 130 100 70 55 40 35 25 15
1 2 3 4 5
Actin

5

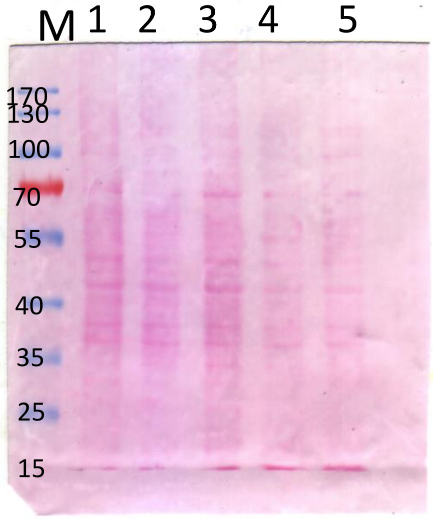
M
1
2
3
4
5
170
130
100
70
55
40
35
25
15

GLUT-4

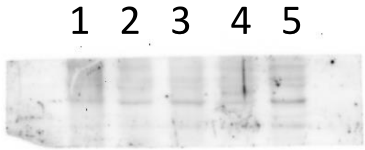
1
2
3
4
5

6

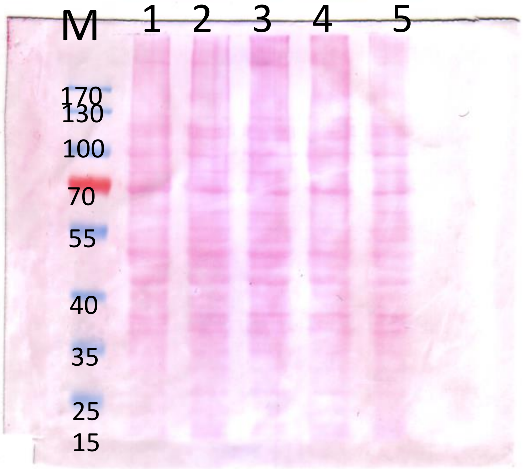
M
1
2
3
4
5
170
130
100
70
55
40
35
25
15

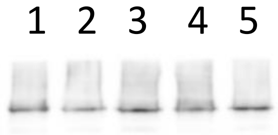
1
2
3
4
5

AKT

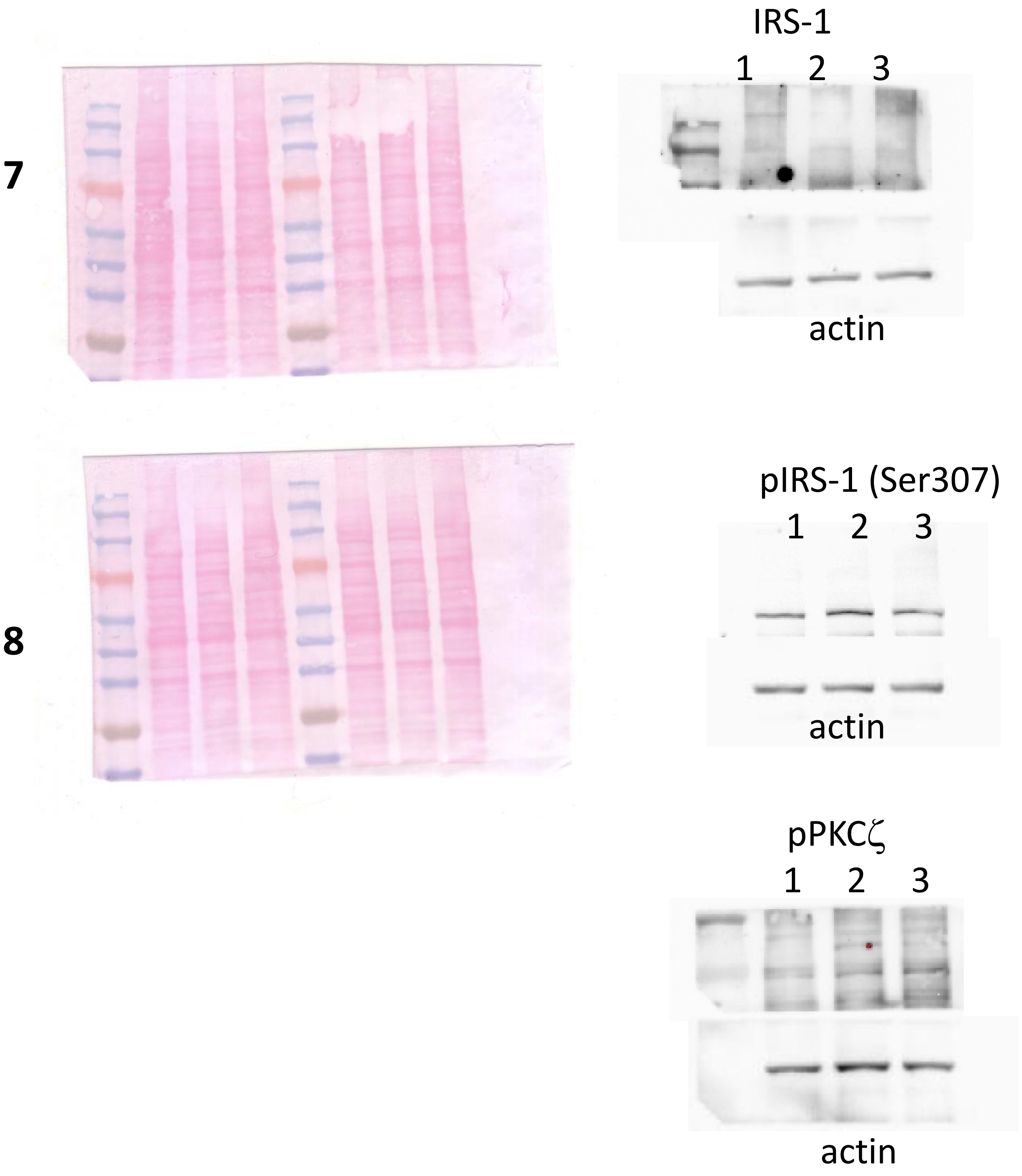

Figure S2: Left: Images of the whole protein blots stained with Ponceau S and showing the molecular weight marker. Right: Images of the corresponding immunoblots whose trimmed inserts have been used for the panel in fig. 4. For blots 1-6: Control = sample 1, insulin = sample 2, GLE = sample 5. For blots 7-8: Control = sample 1, insulin = sample 2, GLE = sample 3.
